# Association of traditional Chinese medicine therapy and the risk of dementia in patients with hypertension: a nationwide population-based cohort study

**DOI:** 10.1186/s12906-017-1677-4

**Published:** 2017-03-29

**Authors:** Kuen-Hau Chen, Ming-Hsien Yeh, Hanoch Livneh, Bor-Chyuan Chen, I-Hsin Lin, Ming-Chi Lu, Tzung-Yi Tsai, Chia-Chou Yeh

**Affiliations:** 1 Department of Chinese Medicine, Dalin Tzuchi Hospital, The Buddhist Tzuchi Medical Foundation, 2 Minsheng Road, Dalin Township, Chiayi, 62247 Taiwan; 2 School of Post-Baccalaureate Chinese Medicine, Tzu Chi University, 701 Jhongyang Road Section 3, Hualien, 97004 Taiwan; 3 School of Chinese Medicine, China Medical University, Taichung, 40402 Taiwan; 4 Rehabilitation Counseling Program, Portland State University, Portland, OR 97207-0751 USA; 5 Division of Allergy, Immunology and Rheumatology, Dalin Tzuchi Hospital, The Buddhist Tzuchi Medical Foundation, 2 Minsheng Road, Dalin Township, Chiayi, 62247 Taiwan; 6 School of Medicine, Tzu Chi University, 701 Jhongyang Road Section 3, Hualien, 97004 Taiwan; 7 Department of Medical Research, Dalin Tzuchi Hospital, The Buddhist Tzuchi Medical Foundation, 2 Minsheng Road, Dalin Township, Chiayi, 62247 Taiwan; 8 Department of Environmental and Occupational Health, College of Medicine, National Cheng Kung University, 138 Sheng-Li Road, Tainan, 70428 Taiwan; 9 Department of Nursing, Tzu Chi University of Science and Technology, 880 Chien-Kuo Road Section 2, Hualien, 97004 Taiwan

**Keywords:** Cohort study, Dementia, Traditional Chinese medicine, Hypertension

## Abstract

**Background:**

Patients with hypertension (HTN) reportedly have a higher risk of developing dementia. However, it remains unclear if use of Traditional Chinese Medicine (TCM), the most common form of complementary and alternative medicine, can help lower the risk of dementia for these patients. So the aim of the study was to investigate the effects of TCM on dementia risk among patients with hypertension.

**Methods:**

This longitudinal cohort study used the Taiwanese National Health Insurance Research Database (NHIRD) to identify 143,382 newly diagnosed hypertension patients aged 20–90 years who received treatment between 1998 and 2007. Among them, 52,365 (36.52%) had received TCM after the onset of hypertension (TCM users), and the remaining 91,017 patients (63.48%) were designated as a control group (non-TCM users). All enrollees were followed until the end of 2012 to record the incidence of dementia. A Cox proportional hazards regression model was used to compute the hazard ratio (HR) of dementia in patients who received TCM.

**Results:**

During the 15-year follow-up, 3933 TCM users and 10,316 non-TCM users developed dementia, representing an incidence rate of 8.41 and 11.55%, respectively, per 1000 person-years. TCM users had a significantly reduced risk of dementia compared to non-TCM users (adjusted HR = 0.76; 95% confidence interval [CI] = 0.74–0.81). The predominant effect was observed among those treated with TCM longer than 180 days (adjusted HR = 0.65; 95% CI = 0.62–0.69). Among the commonly used TCM products, Tian-Ma-Gou-Teng-Yin, Dan-Shen (*Radix Salviae Miltiorrhizae*), Chuan-Niu-Xi (*Radix Cyathulae*), Ge-Gen (*Radix Puerariae*), Jia-Wei-Xiao-Yao-San, and Jue-Ming-Zi (*Semen Cassiae*) were significantly associated with a lower risk of dementia.

**Conclusions:**

Results from this population-based study support the effects of TCM on reducing dementia risk, which may provide a reference for dementia prevention strategies.

## Background

Dementia is characterized by impairment of cognition involving learning, memory, language, executive function, complex attention, perceptual-motor skills, and social cognition [1]. In 2015, 46.8 million people worldwide had dementia, and 9.9 million new cases are reported every year [2]. The occurrence and irreversibility of this illness pose a heavy burden on society and families. The estimated global costs of dementia care have increased from $604 billion in 2010 to $818 billion in 2015 (in US dollars), representing an increase of 35.4% [2]. Although acetylcholinesterase inhibitors and N-methyl-D-aspartate (NMDA) receptor antagonists have been effective for enabling patients to maintain global function, there is currently no treatment to halt or reverse the degenerative progression of dementia [3].

Nowadays, Traditional Chinese Medicine (TCM) has become increasingly popular as an alternative treatment for several critical illnesses, such as diabetes mellitus [4], atopic dermatitis [5], cancer [6], fractures [7], or vertigo [8]. Although previous research has indicated that several TCM herbs have shown potential benefits for dementia intervention [9], the evidence of long-term effects of TCM on dementia risk is still limited.

Hypertension (HTN), a common chronic disorder, is a critical challenge in global public health. It is noteworthy that HTN has been consistently associated with an increased risk of dementia in most cross-sectional and longitudinal cohort studies, and appears to be more relevant when it presents in middle age rather than later in life [10–12]. Results of previous studies have demonstrated that TCM can lower blood pressure and modify vascular risk factors in patients with HTN [13, 14], which may be related to dementia [15, 16]. However, to our knowledge, there is a paucity of information on the benefit of TCM for dementia risk, in particular for subjects with HTN. To address this concern, we studied claims data from the Taiwanese National Health Insurance Research Database (NHIRD) to determine the effect of TCM services on subsequent risk of dementia among HTN individuals. The results of this study may serve as a reference for further pharmacological studies and clinical trials.

## Methods

### Data source

This retrospective cohort study used Taiwan’s Longitudinal Health Insurance Database (LHID), which is maintained by the National Health Insurance Administration and available to Taiwanese researchers. In 1995, Taiwan launched a single-payer national health insurance program designed to remove financial barriers to medical care for all legal residents. As of 2010, over 99% of Taiwan’s population have been enrolled in this program [17]. The LHID, a sub-dataset of the NHI program, is made up of data from 1 million randomly sampled Taiwanese citizens alive in 2000. We collected all medical records available for these individuals from 1997 to 2012. Because a multistage stratified systematic sampling method was used, no statistically significant differences in sex or age existed between the 1 million insured individuals and the general population [17]. This database contains all NHI enrollment files, claims data, and the registry for prescription drugs, which thus provides comprehensive pharmacological utilization information for subjects covered by the insurance program. To date, more than 300 published papers have utilized this de-identified secondary data.

This study was conducted in accordance with the Helsinki Declaration, and it was also evaluated and approved by the local Institutional Review Board and Ethics Committee of Buddhist Dalin Tzu Chi Hospital, Taiwan (No. B10004021–1). Since the LHID files contain only de-identified secondary data, the review board waived the requirement for obtaining informed consent from the patients.

### Study population

**Fig. 1:**
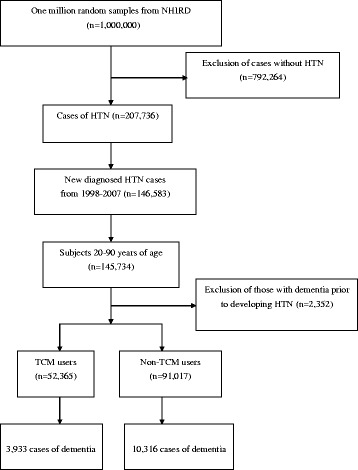
A flowchart about the selection and follow-up of study subjects

The participant selection method is shown in Fig. 1. All diagnoses in this insurance claims data were coded with the *International Classification of Disease, 9th Revision, Clinical Modification* (*ICD-9-CM*). With it, we identified patients 20 to 90 years of age with newly diagnosed HTN within the 1998–2007 time period (*ICD-9-CM* codes: 401–405) as the study cohort. To reduce concern about disease misclassification, we selected only those cases where at least three diagnoses were made during outpatient visits or cases where patients were admitted to a hospital with a primary diagnosis of HTN within the observational period (*n* = 145,734). Patients with HTN who were followed for less than 3 months or who had a prior diagnosis of dementia before the first-time HTN diagnosis were excluded (*n* = 2352). Patients with dementia were identified if they had at least two treatment claims for dementia during outpatient visits or if they had been hospitalized with dementia for *ICD-9-CM* codes 290, 294, or 331. A final group of 143,382 subjects with HTN were included in the data analysis.

In Taiwan, only certified Chinese medicine physicians are entitled to provide TCM services. We used the frequency of visits for TCM to verify the TCM exposure of each study subject. HTN subjects who received TCM for more than 30 days were considered TCM users, whereas those treated for 30 days or less were considered non-TCM users [8]. The index date of the follow-up period for HTN subjects who were classified as non-TCM users was assigned to the date of the first HTN diagnosis, whereas the index date of follow-up period for HTN cases with TCM usage was assigned to the first date of the initiation of TCM services. The end date of the follow-up period for both groups was assigned as the date of the earliest of one of the following: a diagnosis of dementia, the date of withdrawal from the insurance program, or the date of December 31, 2012.

### Demographic characteristics and comorbidity

Demographic characteristics considered in this study included age, gender, income for estimating insurance payment, the availability of TCM resources, and urbanization level of the subject’s residential area. The subjects’ monthly incomes were stratified into 3 levels: ≤ New Taiwan Dollar (NTD) 17,880, NTD 17,881–NTD 43,900, and ≥ NTD 43,901. Urbanization levels were divided into 3 strata: urban (levels 1–2), suburban (levels 3–4), and rural (levels 5–7) areas. Level 1 refers to the “most urbanized” and level 7 refers to the “least urbanized” communities [18]. The comorbid medical conditions, for each individual, were evaluated by using the Charlson-Deyo comorbidity index (CCI) [19]. This index is a widely used method of categorizing comorbidities of patients based on the ICD-9 diagnosis codes found in administrative data. In this study, only diagnoses given ahead of and in concurrence with the diagnosis of HTN were regarded as underlying comorbidities.

### Statistical analysis

We used the chi-square test and Student’s *t*-test to examine the differences in demographic characteristics and comorbidities between subjects who received and those who did not receive TCM services. Thereafter, the Cox proportional hazards regression analysis was applied to compute the hazard ratio (HR) with 95% confidence intervals (CI) of dementia risk in association with TCM use. To test the robustness of the relationship between TCM use and dementia risk, we divided the TCM users into two subgroups: one group used TCM for 30 to 180 days, and the other group used TCM for more than 180 days. Furthermore, a stratified analysis by age and gender using Cox proportional hazards regression was also conducted to assess the relative risk of dementia among the subjects who received and did not receive TCM services. The proportional-hazards assumption was verified using plots of log (−log (survival function) vs log (time) and Schoenfeld residuals vs time. All analyses were conducted using SAS version 9.3 (SAS Institute Inc., Cary, NC, USA), at a *P* < 0.05 statistically significant level.

## Results

We identified 143,382 patients with HTN during the period from 1998 to 2007. Of these, 52,365 subjects received TCM services and 91,017 subjects were classified as non-TCM users. Table 1 shows the basic characteristics of the two groups. Compared to the non-TCM users, HTN patients receiving TCM services were more likely to be female and younger, to have lower monthly incomes and tobacco use, to have higher CCI scores, and to reside in an urban area (all *P* < 0.01).

**Table 1:** Demographic data and selected comorbidities of the study subjects

Variables	Non-TCM users	TCM users	*P-*value
	*n* = 91,017 (%)	*n* = 52,365 (%)	
Age (yr)			<0.001
≤50	22,971 (25.2)	16,461(31.4)	
>50	68,046 (74.8)	35,904 (68.6)	
Mean (SD)	59.74 (13.6)	57.19 (12.4)	<0.001
Gender			<0.001
Female	40,794 (44.8)	29,597(56.5)	
Male	50,223(55.2)	22,768 (43.5)	
Monthly income			<0.001
Low	39,976(43.9)	21,506 (41.1)	
Median	46,352 (50.9)	28,123 (53.7)	
High	4689 (5.2)	2736 (5.2)	
Residential area			<0.001
Urban	49,720(54.6)	29,629 (56.6)	
Suburban	14,209(15.6)	8426 (16.1)	
Rural	27,088(29.8)	14,310 (27.3)	
Tobacco use			0.002
Yes	352 (0.4)	150 (0.3)	
No	90,665 (99.6)	52,215 (99.7)	
CCI			<0.001
Mean (SD)	3.50 (4.42)	4.23 (7.03)	

*TCM* Traditional Chinese Medicine, *SD*, standard deviation; *CCI* Charlson-Deyo comorbidity index

Among the 143,382 HTN subjects, 14,249 first episodes of dementia occurred; 10,316 were reported among the non-TCM users and 3933 among the TCM users during the follow-up of 892,960.27 and 467,500.55 person-years (PYs), respectively. The incidence rate of dementia was lower among TCM users than among non-TCM users (8.41 vs 11.55, respectively, per 1000 PYs), with an adjusted HR of 0.76 (95% CI = 0.74–0.81) (Table 2). Of note, using TCM services for more than 180 days was associated with a 35% decreased risk of dementia among HTN patients (95% CI = 0.62–0.69).

**Table 2:** Risk of dementia for HTN subjects with and without TCM

Patient group	Event	PYs	Incidence	Crude HR (95% CI)	Adjusted HR^a^ (95% CI)
Non-TCM users	10,316	892,960.27	11.55	1	1
TCM users	3933	467,500.55	8.41	0.73(0.71–0.76)	0.76 (0.74–0.81)
TCM use within 30–180 days	2706	289,829.43	9.34	0.82 (0.78–0.85)	0.86 (0.82–0.90)
TCM use for more than 180 days	1277	177,671.13	6.91	0.60 (0.57–0.64)	0.65 (0.62–0.69)

*TCM* Traditional Chinese Medicine, *PYs* per 1000 person-years, *HR* hazard ratio, *CI* confidence interval

^a^Model adjusted for age, gender, urbanization level, monthly income, and CCI scores

As for the sex-specific risk of dementia, we discovered that both female and male HTN patients receiving TCM services had a significantly decreased risk of dementia, with an adjusted HR of 0.71 (95% CI = 0.68–0.74) and 0.84 (95% CI = 0.81–0.91), respectively (Table 3). Additionally, because a significant interaction of age and sex in relation to TCM use occurred, we further performed a stratified analysis by age and sex to determine the effect of TCM on dementia risk. In general, use of TCM was associated with a lower risk of dementia, irrespective of sex. The multivariable stratified analysis verified significant associations of dementia risk with TCM use among male subjects with HTN aged ≤50 years (adjusted HR = 0.72; 95% CI = 0.57–0.89); for female subjects, the more beneficial effect of TCM on the risk of dementia was noted for those older than 50 years (adjusted HR = 0.70; 95% CI = 0.67–0.75) (Table 3).

**Table 3:** Incidence and dementia risk for HTN patients with and without TCM in the stratification of sex and age

Variables	Non-TCM users			TCM users			Crude HR (95% CI)	Adjusted HR (95% CI)
	Case	PYs	Incidence	Case	PYs	Incidence		
Female								
≤ 50 yr	148	81,138.18	1.82	111	79,715.46	1.39	0.77 (0.60–0.98)	0.76^a^ (0.59-0.97)
> 50 yr	4949	320,644.53	15.43	2104	188,214.45	11.18	0.73 (0.70–0.77)	0.70^a^ (0.67-0.75)
All	5097	401,782.71	12.69	2215	267,926.61	8.27	0.66 (0.63–0.69)	0.71^b^ (0.68-0.74)
Male								
≤ 50 yr	307	139,732.62	2.20	107	65,359.01	1.64	0.75 (0.59–0.93)	0.72^a^ (0.57-0.89)
> 50 yr	4912	351,444.93	13.98	1611	134,211.63	12.00	0.85 (0.81–0.91)	0.87^a^ (0.83-0.92)
All	5219	491,177.56	10.63	1718	199,570.64	8.61	0.81 (0.77–0.86)	0.84^b^ (0.81-0.91)

*TCM* Traditional Chinese Medicine, *PYs* person-years, *HR* hazard ratio, *CI* confidence interval

^a^Model adjusted for urbanization level, monthly income, and CCI scores

^b^Model adjusted for age, urbanization level, monthly income, and CCI scores

The most commonly prescribed TCMs for patients with HTN are summarized in Table 4. Of the 15 most common TCMs, 6 were herbal formulas and 9 were single herbs. Tian-Ma-Gou-Teng-Yin (TMGTY) was the most used herbal formula, followed by Dan-Shen and Gou-Teng-San. Of these TCMs, Tian-Ma-Gou-Teng-Yin, Dan-Shen, Chuan-Niu-Xi, Ge-Gen, Jia-Wei-Xiao-Yao-San, and Jue-Ming-Zi were found to be significantly related to a lower risk of dementia (Table 5).

**Table 4:** Top 15 commonly prescribed TCMs for treating HTN during study period

TCM name	Ingredients or generic name	Functions in TCM	Frequency of prescriptions *N* = 503,567 (%)	Average daily does (g)	Average duration of prescription (day)
Tian-Ma-Gou- Teng-Yin	*Rhizoma Gastrodiae; Ramulus Uncariae Cum Uncis; Concha Haliotidis; Fructus Gardeniae Jasminoidis; Radix Scutellariae Baicalensis; Radix Cyathulae; Cortex Eucommiae Ulmoidis; Herba Leonuri; Herba Taxilli; Caulis Polygoni Multiflori; Sclerotium Poriae Cocos*	Calms the Liver, Extinguishes Wind, Clears Heat, Invigorates the Blood, Tonifies the Liver and Kidneys	24,823(4.9)	7.2	11.0
Dan-Shen	*Radix Salviae Miltiorrhizae*	Activates the Blood and dispels Blood Stasis, Cools the Blood and reduces abscesses, Nourishes the Blood and calms the Spirit	13,197(2.6)	3.0	11.7
Gou-Teng-San	*Ramulus Uncariae Cum Uncis; Radix Ginseng;* *Sclerotium Poriae Cocos; Sclerotium Poriae Curcum; Radicem Pini; Radix Ophiopogonis;* *Pericarpium Citri Reticulatae;* *Rhizoma Pinelliae Preparatum; Radix Saposhnikoviae* *Flos Chrysanthemi; Gypsum Fibrosum; Rhizoma Zingiberis Recens; Radix Glycyrrhizae*	Clears Heat from the Liver channel, Descends Liver *Yang,* Extinguishes Liver Wind, Transforms Phlegm, Strengthens the Spleen, Enriches fluids	10,043(2.0)	7.4	9.5
Gou-Teng	*Ramulus Uncariae Cum Uncis*	Extinguishes Wind and alleviates spasms, Drains Liver Heat and pacifies Liver Yang, Releases the Exterior	8957(1.8)	2.8	11.2
Xia-Ku-Cao	*Spica Prunellae*	Clears Liver Fire and brightens the eyes, Clears Hot Phlegm and dissipates nodules	8153(1.6)	2.2	11.0
Zhi-Bai-Di- Huang-Wan	*Radix Rehmanniae Preparata; Fructus Corni Officinalis; Cortex Moutan; Rhizoma Dioscoreae Oppositae; Sclerotium Poriae Cocos; Rhizoma Alismatis Orientalis; Rhizoma Anemarrhenae Asphodeloidis; Cortex Phellodendri Chinensis*	Enriches *Yin,* Nourishes the Essence of the Liver and Kidneys, Reduces Deficiency Fire	6123(1.2)	5.1	11.9
Da-Huang	*Radix et Rhizoma Rhei*	Purges Heat, Loosens the bowels, Promotes Blood circulation, and removes Blood Stasis	5932(1.2)	1.1	11.4
Xue-Fu-Zhu- Yu-Tang	*Semen Pruni Persicae; Flos Carthami Tinctorii; Rhizoma Ligustici Chuanxiong; Radix Angelicae Sinensis; Radix Cyathulae; Fructus Aurantii; Radix Paeoniae Rubra; Radix Platycodi Grandiflori; Radix Bupleuri Chinensis; Radix Rehmanniae Glutinosae; Radix Glycyrrhizae Uralensis*	Invigorates the Blood, Dispels Blood Stasis, Spreads Liver *Qi,* Unblocks the channels, Stops pain	5758(1.1)	6.6	11.5
Chuan-Niu-Xi	*Radix Cyathulae*	Invigorates the Blood, Dispels Blood Stagnation, Promotes Urination and Drains Damp, Tonifies Liver and Kidney, Strengthens Tendon	5660(1.1)	2.3	12.4
Ge-Gen	*Radix Puerariae*	Dispels pathogenic factors from the superficial muscles to relieve fever, Promotes the production of body Fluid, Invigorates the Spleen Yang to stop diarrhea	5329(1.0)	1.5	10.7
Qi-Ju-Di- Huang-Wan	*Radix Rehmanniae Preparata; Fructus Corni Officinalis; Rhizoma Dioscoreae Oppositae; Rhizoma Alismatis Orientalis; Sclerotium Poriae Cocos; Cortex Moutan Radicis; Fructus Lycii Chinensis; Flos Chrysanthemi Morifolii*	Enriches Yin, Nourishes the essence of the Liver and Kidneys, Brightens eyes, improves vision, Enriches Blood	4286(0.9)	5.7	13.8
San-Qi	*Radix Notoginseng*	Arrests bleeding, Resolves Blood Stasis, Promotes the circulation of Blood	4254(0.8)	3.3	10.7
Tian-Ma	*Rhizoma Gastrodiae*	Stops Wind to relieve convulsion, Soothes the Liver, and suppresses hyperactive Liver Yang	4192(0.8)	1.6	11.1
Jia-Wei-Xiao- Yao-San	*Radix Bupleuri Chinensis; Radix Angelicae Sinensis; Radix Paeoniae Lactiflorae; Rhizoma Atractylodis Macrocephalae; Sclerotium Poriae Cocos; Radix Glycyrrhizae Uralensis; Cortex Moutan Radicis; Fructus Gardeniae Jasminoidis; Herba Menthae Haplocalycis; Rhizoma Zingiberis Recens*	Pacifies the Liver, Spreads Liver *Qi,* Strengthens the Spleen, Nourishes the Blood and *Yin,* Regulates menstruation, Sedates the Heart, Clears Heat	3984(0.8)	7.5	13.3
Jue-Ming-Zi	*Semen Cassiae*	Clears Liver Heat, Improves eyesight, Moistens the Intestines to relieve constipation	3949(0.8)	2.1	10.2

**Table 5:** Risk of dementia in relation to the top 15 commonly used TCMs

TCM name	Crude HR (95% CI)	Adjusted HR^j^ (95% CI)
Tian-Ma-Gou-Teng-Yin ^a,c,d,e,f,g,h,i^	0.77 (0.68–0.86)	0.82 (0.76–0.95)^k^
Dan-Shen ^a,d,e,f,g,h,i^	0.71 (0.61–0.83)	0.80 (0.69–0.92)^k^
Gou-Teng-San	0.78 (0.65–0.92)	0.90 (0.74–1.07)
Gou-Teng	0.75 (0.61–0.90)	0.89 (0.75–1.08)
Xia-Ku-Cao	0.70 (0.56–0.86)	0.79 (0.60–1.02)
Zhi-Bai-Di-Huang-Wan	0.88 (0.68–1.10)	0.97 (0.76–1.23)
Da-Huang	0.89 (0.67–1.15)	0.92 (0.70–1.20)
Xue-Fu-Zhu-Yu-Tang	0.82 (0.67–0.99)	0.91 (0.75–1.21)
Chuan-Niu-Xi ^e,f,i^	0.73 (0.58–0.92)	0.79 (0.62–0.98)^k^
Ge-Gen ^a,b,f,g,h,i^	0.62 (0.43–0.79)	0.65 (0.52–0.83)^k^
Qi-Ju-Di-Huang-Wan	0.87 (0.68–1.12)	0.85 (0.66–1.09)
San-Qi	0.85 (0.66–1.10)	0.89 (0.69–1.19)
Tian-Ma	0.98 (0.79–1.22)	0.96 (0.77–1.19)
Jia-Wei-Xiao-Yao-San ^a,b,i^	0.70 (0.54–0.91)	0.79 (0.64–0.98)^k^
Jue-Ming-Zi ^a,e,h,i^	0.57 (0.41–0.80)	0.68 (0.49–0.96)^k^

*HR* hazard ratio, *CI* confidence interval

Potential therapeutic effects of the most promising TCMs:

^a^Reduces β-amyloid protein or its toxicity

^b^Reduces Tau protein or its abnormal phosphorylation

^c^Inhibits NMDA receptor

^d^Inhibits acetylcholinesterase

^e^Anti-inflammatory

^f^Antioxidant activity

^g^Anti-apoptosis

^h^Neuroprotective effects

^i^Medicinal benefits in vascular risk factors

^j^Model adjusted for age, gender, urbanization level, monthly income, and CCI scores

^k^Promising TCMs that were significantly related to the lower risk of dementia

## Discussion

To our knowledge, this is the first population-based cohort study to address the influence of TCM on the risk of dementia among patients with HTN. The study results provide more robust findings about the effects of TCM in this patient population, and may allow clinicians to choose the most appropriate treatment for HTN individuals.

Results of this 15-year follow-up study showed that subjects with HTN who received TCM services exhibited a 24% reduced risk of dementia as compared to those who did not receive TCM services. In addition, the use of TCM services for more than 180 days was associated with a 35% decreased risk of developing dementia. Furthermore, the stratified analysis supported that the TCM intervention significantly reduced the risk of dementia in females more than for males, with adjusted HR of 0.71 and 0.84, respectively. One contributing factor might be that Taiwanese females often exhibit better adherence behaviors than males [20], thus preventing their disease from worsening.

The findings of this study also showed that TCM services may result in fewer effects on females younger than 50 years of age compared to males of similar age. We speculate that younger women may benefit from higher estrogen levels, which could decrease serum low-density lipoprotein-cholesterol (LDL-C) levels and curb coronary thrombosis and atherosclerosis by regulating vascular smooth muscle and endothelial cells [21]. This biochemical benefit may lessen the corresponding effect of TCM services. Taken together, these results clearly demonstrate that earlier and longer use of TCM among HTN patients is associated with reduction of dementia risk.

The two principal types of dementia, Alzheimer’s disease (AD) and vascular dementia (VaD), account for about 70 and 15% of cases of dementia, respectively [22]. Reducing the amount of beta-amyloid protein (Aβ) and tau protein in the brain and modulating Aβ toxicity, including inflammatory response, oxidative stress, and neuronal apoptosis, have been considered the most promising therapeutic strategies available for controlling the progression of dementia [23, 24].

In our study, six TCMs were found to be significantly related to a lower risk of dementia, and these have been reported to exert most of the neuroprotective activities mentioned above. Additionally, most of these TCMs have shown medicinal benefits in reducing vascular risk factors of AD and VaD, and thus they may also help reduce the risk of dementia [15, 16]. The possible pharmacological mechanisms of the most common TCMs for the treatment of patients with HTN are summarized in Tables 4 and 5.

We discovered that the most commonly prescribed herbal formula, Tian-Ma-Gou-Teng-Yin (TMGTY), was used in TCM for relieving symptoms related to high blood pressure, such as headaches and dizziness. Results of previous studies have shown that TMGTY has significant effects on a variety of HTN-caused cardiovascular diseases [14]. Recent scientific evidence has also demonstrated that TMGTY provides neuroprotective effects for patients with Parkinson’s disease [25]. TMGTY has also been found to inhibit the NMDA receptor. This leads to a reduction in necrosis and apoptosis in neuronal cells through a variety of pharmacological effects, including anti-inflammatory, antioxidative, and anti-apoptotic activities [26]. Moreover, the components of TMGTY could decrease the activity of acetylcholinesterase and show potent anti-aggregation effects on Aβ proteins [27, 28].

Dan-Shen (*Radix Salviae Miltiorrhizae*) and Ge-Gen (*Radix Puerariae*) are two herbs frequently used for the treatment of angina and other cardiac symptoms in TCM. Recent studies have demonstrated their cardioprotective and anti-atherosclerosis effects. Results of pharmacological studies suggest that these herbs can control high blood pressure, lower serum lipids, and improve microcirculation [29]. Dan-Shen demonstrated neuroprotective effects [29], including inhibition of Aβ aggregation, oligomerization, and fibril formation through upregulation of activity of alpha-secretases [30]. Dan-Shen also acts as an acetylcholinesterase inhibitor [31], and it could improve age-related oxidative stress and inflammatory response [32], protect endothelial cells from hydrogen peroxide damage, and inhibit apoptosis [33]. Ge-Gen was found to show potential medicinal benefits in diabetes and cardiovascular and cerebrovascular diseases [34], and it could alleviate neurological deficits and improve learning and memory after ischemia/reperfusion-induced cerebral microcirculatory disturbances [35]. Recent studies have also demonstrated that Ge-Gen could inhibit Aβ plaque accumulation [36], suppress tau protein expression [37], and antagonize neuronal apoptosis induced by oxidative stress [38].

Chuan-Niu-Xi **(** *Radix Cyathulae*) has demonstrated various beneficial pharmacological activities, including analgesic, immunostimulant, antitumor, anti-inflammatory, and antiaging effects. Its use removes blood stasis, restores menstrual flow, and induces diuresis for treating stranguria. It also has antioxidant qualities [39].

Jia-Wei-Xiao-Yao-San is widely used to relieve emotional and neuropsychological disorders such as depression, stress [40], and dyskinesia in patients with schizophrenia [41]. Researchers have also shown that Jia-Wei-Xiao-Yao-San can reduce tremors of antipsychotic-induced parkinsonism [42], relieve migraine headache [43], and improve the survival rate of type 2 diabetes patients with HTN [44]. Moreover, the components of Jia-Wei-Xiao-Yao-San can inhibit abnormal tau phosphorylation, suppress the release of Aβ peptides, and decrease beta-amyloid-induced neurotoxicity [45, 46].

Another TCM herb, Jue-Ming-Zi (*Semen Cassiae*) has shown neuroprotective effects in animal models of ischemic stroke and Parkinson’s disease [47]. Results of a recent study also demonstrated that this herb can ameliorate amyloid-β-induced synaptic dysfunction through anti-inflammatory and Akt/GSK-3β pathways [48]. In our study, all of the previously mentioned TCM agents relieved the symptoms of HTN and decreased the risk of developing dementia.

Although our study is the first to recently investigate the effect of use of TCM on the risk of dementia among subjects with HTN, there are some important limitations to consider. First, identification of use of TCM and outcomes were based on three categories in the *ICD-9-CM*, and inaccurate diagnosis may have occurred. To minimize this error, we only selected subjects with either HTN or dementia after they were recorded as having at least three outpatient visits reporting consistent diagnoses or one inpatient admission for HTN. It should also be noted that the NHI of Taiwan randomly samples claims from hospitals, interviews patients, and reviews medical charts to verify the accuracy of medical records. Second, we could not account for other confounding factors, such as the use of tobacco and alcohol, physical activity, dietary preferences, social network relationships, coping strategies, or educational level, which were unavailable from the claims data. Further studies controlling for those untested factors are recommended to assess whether the present findings are replicable among other demographically and geographically diverse groups. Third, we were unable to contact the enrolled patients directly about the use of Chinese herbs due to the anonymity of identification numbers in the database. Even so, we were still able to demonstrate the benefit from TCM use. Nearly 95% of dosing frequencies in Chinese herbs are typically only used for one week in clinical practice, so those who continued to receive the same prescriptions for a longer period were therefore likely to have used the prescribed medication [49]. Fourth, the NHI program only pays for TCMs prescribed by Chinese medicine physicians, not over-the-counter TCMs. Therefore, the use of TCM may be underestimated. However, the NHI covers TCM prescriptions (the concentrated herbal powder) manufactured by GMP-certified pharmaceutical companies in Taiwan. Within the NHI program, the copayment for visiting a TCM clinic is only approximately 10 USD, which may greatly enhance the accessibility to TCM services. Fifth, findings from any retrospective cohort study are generally less sound than these from randomized trials because cohort study designs are subject to various biases related to uncontrolled confounding effects. Despite our careful attention to the study design, employing adequate control of confounding factors, unpredictable biases could still remain if they stem from unmeasured or unknown confounders. Notwithstanding these limitations, the strengths of this study must also be acknowledged and these included the immediate availability of data, the comprehensiveness of the database, and the statistical power derived from the samples’ large sizes. In addition, this retrospective 15-year cohort study allowed us to examine in detail the association of TCM usage with the subsequent risk of dementia, and the corresponding findings could serve as a reference for future treatments.

## Conclusions

The results of this population-based, retrospective cohort study show that the use of TCM during treatment of HTN was associated with a 24% lower risk of developing dementia compared to the risk among non-TCM users. TCM users had a significantly reduced risk of dementia compared to non-TCM users (adjusted HR = 0.76; 95% CI = 0.74–0.81). The predominant effect was observed among those treated with TCM longer than 180 days (adjusted HR = 0.65; 95% CI = 0.62–0.69). These findings could serve as a reference for healthcare providers, in helping to establish more effective therapeutic interventions to improve the prognosis of patients with HTN and prevent subsequent dementia.

## Abbreviations

Aβ: beta-amyloid AD Alzheimer’s disease CCI Charlson-Deyo comorbidity index CI Confidence interval GMP Good Manufacturing Practice HR Hazard ratio HTN Hypertension LHID Longitudinal Health Insurance Database NHI National Health Insurance NHIRD National Health Insurance Research Database NMDA N-methyl-D-aspartate NTD New Taiwan Dollar PYs Per 1000 person-years SD Standard deviation TCM Traditional Chinese Medicine TMGTY Tian-Ma-Gou-Teng-Yin USD United States Dollar VaD Vascular dementia
